# Residual Malaria: Limitations of Current Vector Control Strategies to Eliminate Transmission in Residual Foci

**DOI:** 10.1093/infdis/jiaa582

**Published:** 2021-04-27

**Authors:** Mario H Rodriguez

**Affiliations:** Centro de Investigaciones Sobre Enfermedades Infecciosas, Instituto Nacional de Salud Pública, Cuernavaca, Morelos, Mexico

**Keywords:** residual malaria

## Abstract

The transmission of Plasmodium parasites in residual foci is currently a major roadblock for malaria elimination. Human activities and behavior, along with outdoor biting mosquitoes with opportunistic feeding preferences are the main causes of the inefficacy of the main vector control interventions, long lasting insecticide-impregnated nets and insecticide residual spraying. Several strategies to abate or repel outdoor biting mosquito vectors are currently being researched, but the impact of insecticide resistance on the efficacy of these and current indoor-applied insecticides requires further assessment. Understanding the human, ecological and vector factors, determining transmission in residual foci is necessary for the design and implementation of novel control strategies. Vector control alone is insufficient without adequate epidemiological surveillance and prompt treatment of malaria cases, the participation of endemic communities in prevention and control is required. In addition, malaria control programs should optimize their structure and organization, and their coordination with other government sectors.

Since the reactivation and upscaling of malaria interventions in 2000, efficacious treatment with antimalarial drugs and interventions to control mosquito vectors have contributed to the major declines in global malaria morbidity and mortality. Between 2000 and 2015, malaria cases globally declined an estimated 37% and the mortality rate was reduced by 60%. However, in 2016, estimated malaria cases (216 million) were higher than those estimated for 2015 (211 million) [1]. In 2017, the number of cases increased to 219 million with 435 000 deaths [2], while the number of cases in the most endemic African countries increased by 3.5 million, and marginal increases occurred in several countries of the American, South Asian, and Western Pacific regions. The causes of this reverse are not yet documented, but a decline in malaria funding, inadequate malaria control programs, limited antivector interventions, patient treatment, and environmental factors, such as heavy rainfall in India and equatorial Africa, have been implicated [3]. The low financial commitment of policymakers and the poor compliance with antimalaria interventions by endemic communities are also important causes.

On the other hand, the progress represented by 20 countries that have eliminated autochthonous transmission [1] support an optimistic possibility to extend elimination to other endemic areas. However, the persistence of transmission in residual foci is currently a major roadblock in countries approaching malaria elimination.

## MALARIA, PARASITE TRANSMISSION, AND CONTROL INTERVENTIONS

*Plasmodium falciparum* and *P. vivax* (the main agents), the infected and exposed humans, and the vector mosquito populations are the interactive components of the malaria life cycle. Parasite transmission from humans to mosquitoes to humans occurs within specific environmental conditions that support mosquito breeding, survival, and feeding on humans (reviewed by Killeen [4]).

The Rollback Malaria Partnership strategy for malaria control was aimed at universal coverage for all populations at risk in a context of efficient control programs within operational and policy-adaptable, strong health systems [5]. Malaria control measures are aimed at interrupting the parasite life cycle. These include reducing the parasite population by opportune case detection using rapid diagnostic test and microscopy, followed by prompt administration of effective antimalarial drugs, and reducing human-vector contact and abating mosquito populations using antivector measures, such as long lasting insecticide-impregnated nets (LLINs) and indoor insecticide residual spraying (IRS). Concomitantly, environmental management and antimosquito larvae interventions are measures to reduce mosquito populations [6]. The need for the participation of endemic communities in surveillance and the implementation of all the above-mentioned control strategies are also recognized, but this is a difficult task seldom incorporated among the control programs’ objectives.

## MALARIA-ENDEMIC AREAS AND RESIDUAL TRANSMISSION FOCI

The operative definition of a malaria transmission focus of the former World Health Organization (WHO) Malaria Eradication Program refers to locations with defined geographic circumscription, situated in an active or previously active malaria area, where continuous or intermittent epidemiological factors support malaria transmission [7]. This concept, along with the description of the functional status of the foci (active, interrupted, and emergent transmission), was the basis for the epidemiological stratification that guided antimalarial interventions. The inclusion of abiotic (geographic extension and climatic characteristics) and biotic components (human population, parasite species, and their antimalarial susceptibility, as well as information on local mosquito vector bionomics, behavior, and insecticide susceptibility), contributed to a better understanding of the ecology and the interactions of human and mosquito populations that determine malaria parasite transmission in each focus [8]. This concept of malaria foci and the factors that define their epidemiology and parasite transmission are still valid and currently accepted.

Malaria-endemic countries and areas contain numerous foci with diverse conditions that require local adaptations of antimalaria strategies and interventions. The Global Malaria Program considers malaria elimination at country level to progress as a continuum [9]. In this sense, autochthonous transmission would be controlled and then eliminated in foci of easier control, and later progress to more difficult ones, until elimination in the entire endemic area is achieved. However, antimalaria activities could be effective in most parts of a given endemic country but attain variable success in other areas. This variability explains why in some countries, as malaria elimination progresses, residual malaria foci of transmission remain.

A residual malaria focus could be defined as a distinct location where transmission persists despite full coverage with antimalarial interventions that are effective in the rest of the country’s endemic areas. Similar to what occurs in the controlled endemic areas, factors that determine transmission in residual foci vary among localities and times [10]. The extended success of LLINs and IRS interventions is the basis for a commonly used operational definition of residual malaria, that is persisting transmission after full coverage with LLINs and/or IRS containing active ingredients against fully susceptible local vectors [4].

## LIMITATION OF CONTROL ACTIVITIES IN RESIDUAL FOCI

Malaria elimination requires integrated interventions, including enhancing and optimizing vector control and case management, as well as improving surveillance, as core interventions to detect, characterize, and monitor all cases [8, 9]. Assuming an efficient case management (universal access to diagnosis and effective treatment), it is generally assumed that the main cause of malaria persistence is the failure in vector control. This failure could be the result of the inefficient application of the control intervention, insufficient exposure of mosquitos to the applied compounds, or have roots in the acceptance of the communities and their participation in the deployment of the offered interventions [11].

Among the arsenal of strategies to control mosquito vectors, environmental management and antimosquito larvae interventions to reduce mosquito populations have been successfully applied to control and eliminate transmission in extensive areas [6], but these have been ineffective in residual foci. Their application to control extensive areas is limited due to the need for a good understanding of the characteristics and dynamics of the breeding sites of the local vectors, and this is true for residual foci. Since the worldwide reactivation of the malaria control programs in 2000, LLINs and IRS have been the most effective control measures [12]. A direct effect of these interventions is exerted on indoor-feeding mosquitoes, with a preference for humans over animals. Interestingly, although mosquito vectors with a strong preference for human blood are better malaria vectors, feeding indoors renders them more susceptible to LLINs and IRS [13]. Although LLINs were designed to protect humans while sleeping indoors and IRS to kill mosquitoes resting in indoor walls, these have been associated with abatements of vector populations [14], which indicates that they could reduce mosquito densities to a minimum, leading to a collapse of the entire population [14, 15]. This probably explains their success in several malaria areas [16]. However, their inability to stop transmission in malaria residual foci could be due to several reasons, such as the susceptibility of the local vector to the applied insecticide, coverage rates, quality and timing of implementation, and the acceptance of target communities [4, 17].

The increasing physiological resistance of vectors to insecticides has been implicated as a major threat to the use of these interventions [18]. However, some studies have indicated that insecticide resistance might not reduce their capacity to control malaria transmission. For instance, an evaluation carried out in 340 locations in 5 countries revealed that LLINs remained effective control measures, despite pyrethroid resistance in the target mosquito populations [19]. However, more research involving the bionomics and behavior of mosquito vectors and endemic communities is needed to understand how indoor-applied insecticides can attain the reduction of mosquito populations [14, 15] and the impact of insecticide resistance on the conformation of residual malaria foci.

Mosquito feeding and resting behaviors, along with human activities exposing them to outdoor biting, perpetuate residual malaria transmission and are the main limitations for the efficacy of LLIN- and IRS-based interventions [20]. The ability of LLINs and IRS to control malaria vectors depends on the opportunity to act on mosquitoes when they seek or feed on humans. This restricts their success to mainly indoor-biting, anthropophagic mosquitoes. However, opportunistic mosquitoes may seek human hosts if their preferred host is scarce. For instance, *Anopheles funestus* and *An. gambiae,* 2 very efficient African malaria vectors, have been controlled or eliminated using LLINs and IRS [4, 16]. These mosquitoes have strong biting preferences for humans while they are asleep at late hours of the night [21]. However, in the Solomon Islands, these tools were also useful to eliminate *An. punctulatus* and *An. koliensis,* which have preferences to feed on pigs, because of the low availability of these animals [16]. On the other hand, a group of outdoor-biting mosquito species, flexible as to feeding on animals and frequently enough in humans, are responsible for residual malaria transmission in many endemic countries [22]. Examples are *An. arabiensis* and *An. colluzzii* in Africa, *An. dirus* in South-East Asia, *An. farauti* in Oceania, and in the Americas *An. darlingi* [10]. The availability of preferred hosts of *An. arabiensis,* which feeds mainly on cattle but also attacks humans, and of *An. farauti*, with a preference for pigs but also feeds on humans, vary across their geographic distribution [23]. Thus, the efficacy of LLINs and IRS to control these mosquitoes is related to the abundance of livestock [24].

Besides feeding mainly on animals but biting humans and resting outdoors, mosquitoes avoid exposure to indoor-applied insecticides by reducing entry to and early exit from houses [25]. *Anopheles farauti,* which feeds outdoors early in the evening, evades these insecticide measures and *An. darlingi*, a zoophilic species which opportunistically feeds indoors on humans, does not remain inside houses long enough to acquire insecticidal doses [26]. These observations support the usefulness of information about the bionomics and behavior of the vector mosquitoes in the controlled endemic areas and the differences that may explain the poor performance of the interventions against those in residual foci.

Several socioeconomic and cultural characteristics of the endemic communities may affect the efficacy of antimalaria interventions, such as insufficient adherence to treatment [27, 28], high mobility, and low perception of the risk of acquiring the infection [29]. Human risk behaviors include the irregular use of LLINs [30] and outdoors activities that expose people to early- and late-biting vectors [31, 32]. Poor economic conditions have been associated with low use of LLINs [33] and people leaving in poor housing conditions are more susceptible to malaria infection [34]. Economic activities without protection in forests and sleeping in shelters that allow unhindered entry of mosquitoes entry also increase the risk of malaria infection [10, 11]. In addition, as malaria control progresses, communities perceive the reduced number of clinical cases as an indication of low risk for infection, which in turn results in the reduction of the use of antimalaria protection measures [35]. These limiting factors should be considered when assessing the persistence of malaria transmission in residual foci. Although many risky social conditions could only be remediated with socioeconomic improvement, a better understanding of the interaction of endemic populations with their local vectors is needed for informing communities and designing participatory interventions.

## MAIN CHALLENGES TO CONTROL AND ELIMINATE RESIDUAL MALARIA

The Global Vector Control Response 2017–2030 identified several interconnected challenges impeding progress in the control of vector-borne diseases in general, which are also applicable to malaria [36]. These include the competition for resources and insufficient synergy among disease-specific programs, increased population displacements, and political and financial constraints, along with a lack of evidence to support intervention activities.

The elimination of residual malaria requires the adaptation of antimalaria strategies to the epidemiological situations in each residual setting, but methods to identify residual foci are not yet available [37] and guidance is needed for assessing progress [38].

Malaria control programs require better structures and organization to progress towards elimination. In this regard, strengthening structures and improving the organization of health services are the foundation for malaria control and this increases in importance when elimination of residual foci is the objective. Assessing the persistence of malaria transmission in residual foci requires the identification of a particular risk factor. Strengthening control programs should enable them to adapt interventions to the biology, ecology, and behavior of vector mosquitoes, as well as to identify the particular situations that expose humans to mosquito bites. Accordingly, entomological studies to assess the participation of local anophelines in transmission should be part of integrated strategies.

Community engagement is an integral component of primary health care and is required for effective interventions of integrated control approaches [39, 40]. However, the participation of communities and community health workers is seldom included in malaria control strategies and often circunscrubed to to diagnosis and treatment [41]. Implementation research is needed to guide strategies for structured participation of communities and, in particular, community health workers in designing and implementing prevention and control measures. Community health workers could participate in addressing misconceptions of malaria risk and increasing the communities’ awareness of behaviors that expose humans to vector mosquito bites. This is of particular importance in residual transmission foci [42].

## NEW STRATEGIES AND TOOLS

The limitations of LLINs and IRS to control the vectors responsible for outdoors transmission is a major limitation for the elimination of residual foci. The Global Malaria Program advises improving the implementations of the currently available tools and focuses on assessing the practicality, effectiveness, and affordability of new strategies. It also recommends that these strategies be based on protection against outdoor-biting mosquitoes and the reduction of vector populations responsible for outdoors transmission [43]. It is implicit that the implementation of current and new potential strategies to control mosquito vectors in residual foci requires a clear understanding of the vector bionomics, behavior, and interactions with the human population.

It is recognized that substantial abatements of these populations, rather than personal protection, is required to stop outdoors transmission [10]. Nevertheless, vapor-phase insecticides used to repel outdoors biting [44] have demonstrated deleterious and even killing effects on mosquitoes [10] and reductions in malaria transmission [45].

New strategies for abating outdoor-biting mosquito populations are currently being investigated, including killing mosquitoes when they feed on sugar sources and on livestock, reviewed in Killeen et al and Barreaux et al [46, 47]. A trial using mosquito traps demonstrated effective abatement of *An. funestus* and reduction of malaria transmission in western Kenya [48]. Additionally, exposure to toxic products in attractive sugar baits has proven effective to abate *An. sergenty* in the Jordan Valley [49] and *An. gambiae* in Mali [50].

Several strategies targeting domestic animals could be used to control mosquitoes that have an opportunistic feeding behavior [51]. The application of insecticides to cattle was associated with a reduction of the survival of *An. arabiensis* in western Kenya [52] and of *An. stephensi* and *An. culicifacies* in Pakistan, with a reduction of the numbers of *P. falciparum* and *P. vivax* clinical cases [53]. In the same way, insecticides applied to corrals and livestock shelters has been proposed as an extension of indoor-applied insecticides [54].

The use of systemic veterinary insecticides (endectocides) to control malaria vectors has attracted much interest among malaria researchers [55]. These compounds, circulating in the blood of treated cattle, kill or reduce the survival of mosquitoes that feed on these animals. For instance, reduced survival was observed in *An. arabiensis* that fed on cattle and humans treated with ivermectin [56, 57].

In conclusion, the persistent transmission of *Plasmodium* parasites in residual foci is currently a major roadblock for malaria elimination in countries approaching malaria elimination. Human activities and behavior, along with outdoor-biting mosquitoes with flexible feeding preferences, are the main causes of the inefficacy of the main vector control interventions, LLINs and IRS. Several strategies to abate or repel outdoor-biting mosquito vectors are currently being researched, but the impact of insecticide resistance on the efficacy of these and current indoor-applied insecticides requires further assessment. Understanding the human, ecological, and vector factors determining transmission in residual foci is indispensable for the design and implementation of vector control strategies. Vector control alone is insufficient without adequate epidemiological surveillance and detection and prompt treatment of malaria cases detected, the participation of the communities in prevention and control is required, and malaria control programs should optimize their structure and organization, as well as their coordination with other government sectors.

## Notes

***Supplement sponsorship***. The supplement is sponsored by TDR, the Special Programme for Research and Training in Tropical Diseases, based at the World Health Organization.

***Potential conflicts of interest.*** The author reports no conflicts of interest. The author has submitted the ICMJE Form for Disclosure of Potential Conflicts of Interest. Conflicts that the editors consider relevant to the content of the manuscript have been disclosed.
